# The Mother–Offspring Conflict: The Association Between Maternal Sleep, Postpartum Depression, and Interbirth Interval Length

**DOI:** 10.1177/14747049211046162

**Published:** 2021-10-12

**Authors:** Annika Gunst, Elin Sjöström, My Sundén, Jan Antfolk

**Affiliations:** 1 Department of Psychology, 1040 Åbo Akademi University, Turku, Finland

**Keywords:** mother-offspring conflict, infant night waking, maternal sleep, postpartum depression, interbirth intervals, sleeping problems, parent-offspring conflict, parental investment

## Abstract

To test the hypothesis that infant night waking is an adaptation to increase interbirth intervals (IBIs) (i.e., the time between a mother’s consecutive births) by exhausting the mother, we made an initial attempt at investigating whether maternal sleep disturbance is associated with longer IBIs. We also explored whether postpartum depression symptoms mediated the association between maternal sleep disturbance and IBI length. We used retrospective self-reports from 729 mothers living in Finland. We conducted structural regressions separately for the mother’s two first children at two different age intervals (0–1 and 1–3 years). Infant night waking was associated with maternal sleep disturbance (β  =  .78–.84) and maternal sleep disturbance was associated with postpartum depression symptoms (β  =  .69–.81). Postpartum depression symptoms were also associated with longer IBIs for the first child (β  =  .23–.28). This result supports the notion that postpartum depression in and of itself could be viewed as adaptive for the offspring’s fitness, and not just as an unintentional byproduct of the mother’s sleep disturbance. Contrary to our prediction, maternal sleep disturbance was, however, associated with shorter IBIs for the first child (β  =  −.22 to −.30) when including postpartum depression symptoms in the model. We discuss the potential role of social support as an explanation for this unexpected result.

## Introduction

According to the mother-offspring conflict theory, what is the ideal amount of maternal investment from the perspective of the infant is higher than from the perspective of the mother (Trivers, 1972). The introduction of siblings can decrease the mother’s investment of her limited resources in any one infant. Since the infant has 100% of its own genes but shares only 50% with its siblings, the infant’s fitness benefits more from investment in itself compared to investment in any siblings. For the mother’s fitness, it is, on the other hand, better to spread the investment equally between all current and potential future offspring.

One way mother-offspring conflict might express itself is in the length of interbirth intervals (IBIs; the time between a mother’s consecutive births). The optimal IBI differs from the standpoint of the mother’s and the offspring’s fitness, with the mother benefitting more from shorter intervals than the offspring (Haig, 2014). This is because human females are fertile during a limited time-span, and shorter IBIs increase the mother’s chances of maximizing her reproductive success, thus maximizing her fitness (Jones et al., 1987). For the offspring, however, longer IBIs decrease sibling rivalry over maternal investment, benefitting the child’s fitness. Thanks to modern healthcare, IBI length is no longer as predictive of offspring survival (Haig, 2014). Still, in hunter-gatherer societies, longer IBIs correlate with a reduction in mortality rates for the offspring. For instance, for the !Kung hunter-gatherers in Southern Africa, IBIs of two years resulted in a mortality rate of 70%, with the rate decreasing to 10% for IBIs of four years (Jones et al., 1987). For the Sereer in rural Senegal, IBIs of less than two years resulted in a mortality rate of 16%, whereas for IBIs longer than two years the mortality rate was 4% (Ronsmans, 1996). Subsequently, it is in the offspring’s favor to promote longer IBIs than what is optimal for the mother.

Previous studies have suggested that infant night waking to breastfeed could be an adaptive strategy by the offspring to lengthen the IBI by suppressing the mother’s fertility (Haig, 2014; Jones et al., 1987). A recent study by Gunst et al. (2021) suggested that infant night waking could be an adaptation to lengthen the IBI by exhausting the mother through disturbed sleep. The authors hypothesized that mothers exhausted by their offspring would be less likely to conceive and gestate additional offspring (e.g., by decreasing the mother’s sex drive, De Judicibus & McCabe [2002]; or by decreasing her fertility by hypothalamic-pituitary-adrenal activation, White [2016]), consequently extending the IBI. Moreover, according to Gunst et al. (2021), postpartum depression symptoms could in this case be a result of mother-offspring conflict. Bearing in mind the negative impact that postpartum depression has for both the mother’s and the infant’s fitness (Slomian et al., 2019), it is from an evolutionary perspective hard to grasp why it is so prevalent, affecting up to 15% of mothers (Gavin et al., 2005; Shorey et al., 2018). Yet, if infant night waking is an adaptation to increase the length of the IBI by exhausting the mother, then postpartum depression could be an unintentional byproduct of this behavior (Gunst et al., 2021). Alternatively, postpartum depression could be adaptive from the offspring’s fitness’ point of view if it makes the mother less able to conceive and gestate an additional offspring, thus lengthening the IBI.

It is unsurprising that frequent infant night waking affects maternal sleep. For instance, a recent German population-based large-scale longitudinal study showed that parental sleep satisfaction declines postpartum, with maternal sleep being especially affected (Richter et al., 2019). Studies on adult sleep disturbance have also consistently associated sleeping problems with poorer mental health as well as poorer motor and cognitive functioning (Bayer et al., 2007; Belenky et al., 2003; Dinges et al., 1997; Pilcher & Huffcutt, 1996; Van Dongen et al., 2003). Furthermore, studies have shown that the stress resulting from sleep deprivation can make people more vulnerable for mood-related disorders such as dysphoria and mood lability (Bayer et al., 2007; Dinges et al., 2005; Riemann et al., 2001; Ross et al., 2005) and changes in sleep patterns are also a common symptom of a major depressive disorder (American Psychiatric Association, 2013). Moreover, when infant sleep treatments decrease the number of night wakings, maternal postpartum depression symptoms usually decrease as well (Armstrong et al., 1998; Hiscock & Wake, 2001), suggesting that sleep problems make mothers more prone to develop postpartum depression. However, the relationship between maternal sleep disturbance, postpartum depression symptoms, and IBI length remains largely unexplored.

### Aims

Based on the premise that the infant benefits more from longer IBIs than the mother and that infant night waking is an adaptation to increase IBIs by exhausting the mother, we examined whether maternal sleep disturbance is associated with longer IBIs and postpartum depression symptoms. We were specifically interested in testing whether more maternal sleep disturbance was associated with longer IBIs. We also explored whether this association would be mediated by postpartum depression symptoms. In line with the substantial literature on maternal sleep, we also expected that more infant night waking would be associated with more maternal sleep disturbance and that more maternal sleep disturbance would be associated with more postpartum depression symptoms.

## Method

### Ethical Statement

The current study was granted ethical permission by the Board for Research Ethics at Åbo Akademi University before data collection began. All participants gave their informed consent prior to participating in the study. They were informed that participation is voluntary and that they can terminate their participation at any point during the survey.

### Participants and Procedure

We collected data in April 2020. We recruited mothers between ages 18–60 with at least two biological children. Mothers of twins were excluded from the study. The participants were recruited through a Facebook advertisement targeting female adults living in Finland. After completing the survey, participants were invited to a separate questionnaire where they could fill out their e-mail address to participate in a lottery of a 100€ gift card to a multi-brand online shop. The completion rate for those who started the survey was 64.0%. Participants who did not respond to all items in the questionnaire were excluded from the statistical analyses. The final sample consisted of 729 mothers, aged 22 to 60 years old, with a mean age of 41.3 years (*SD*  =  9.2).

### Measures

All participants were asked to provide some demographic information as well as descriptive information regarding the IBIs, consisting of questions about breastfeeding, use of contraceptives, miscarriage, abortion, and in-vitro fertilization. Mothers of two children answered the descriptive information and below-mentioned measures for the first and only IBI (i.e., the period between the first and the second birth). We assessed the below-mentioned measures separately for two age intervals; the child’s first year and when the child was between 1 and 3 years old. Mothers of three or more children additionally answered the descriptive information and below-mentioned measures for the second IBI (i.e., the period between the second and third birth).

**Infant night waking.** To measure the frequency of infant night waking, we used three self-made questions: “*How many times on average did your child wake up per night?”*, “*How well did your child sleep?”* and “*How difficult was it to get your child to sleep through the night?”*. The items were rated on a 5-point Likert-type scale (1–5), with the anchors 1  =  *hardly ever/very well/very easy*; 5  =  *more than five times per night/very poorly/very hard* (i.e., higher scores indicating more wakings).

**Maternal sleep disturbance.** To measure the level of maternal sleep disturbance, we used the Insomnia Severity Index (ISI; Morin, 1993). The ISI is considered a valid and reliable self-report questionnaire consisting of seven items that are designed to determine the severity of insomnia-related issues taking place during the night as well as during the day (Bastien et al., 2001). Each item on the index is rated on a 5-point Likert Scale (0–4), with higher scores indicating more problems. The total score on the index ranges from 0 to 28, with 0–7 indicating absence of insomnia, 8–14 sub-threshold insomnia, 15–21 moderate insomnia, and 22–28 severe insomnia (Morin et al., 2011). In addition to the ISI, we used one self-made question measuring average hours of sleep: “*How many hours on average did you sleep per night?”*. We set the outliers to 12 and reverse-coded the item, giving it a range of 0–12 with higher scores indicating more problems.

**Postpartum depression.** To measure symptoms of postpartum depression, we used the seven-item short version of the Edinburgh Postnatal Depression Scale (EPDS; Cox et al., 1987; Gollan et al., 2017). The original version is a standardized self-report questionnaire consisting of 10 items assessing the components of postpartum depression during and after pregnancy (Cox et al., 1987). The seven items that were used for this study (items 1, 2, 6–10) focus solely on measuring symptoms of depression, with the three excluded items measuring anxiety (Gollan et al., 2017). Each item on the scale is rated on a 4-point Likert Scale (0–3) with the total score ranging from 0 to 21. A higher total score indicates more depressive symptoms, with scores above eight having been suggested as a threshold for depression.

### Statistical Analyses

**Figure 1.**
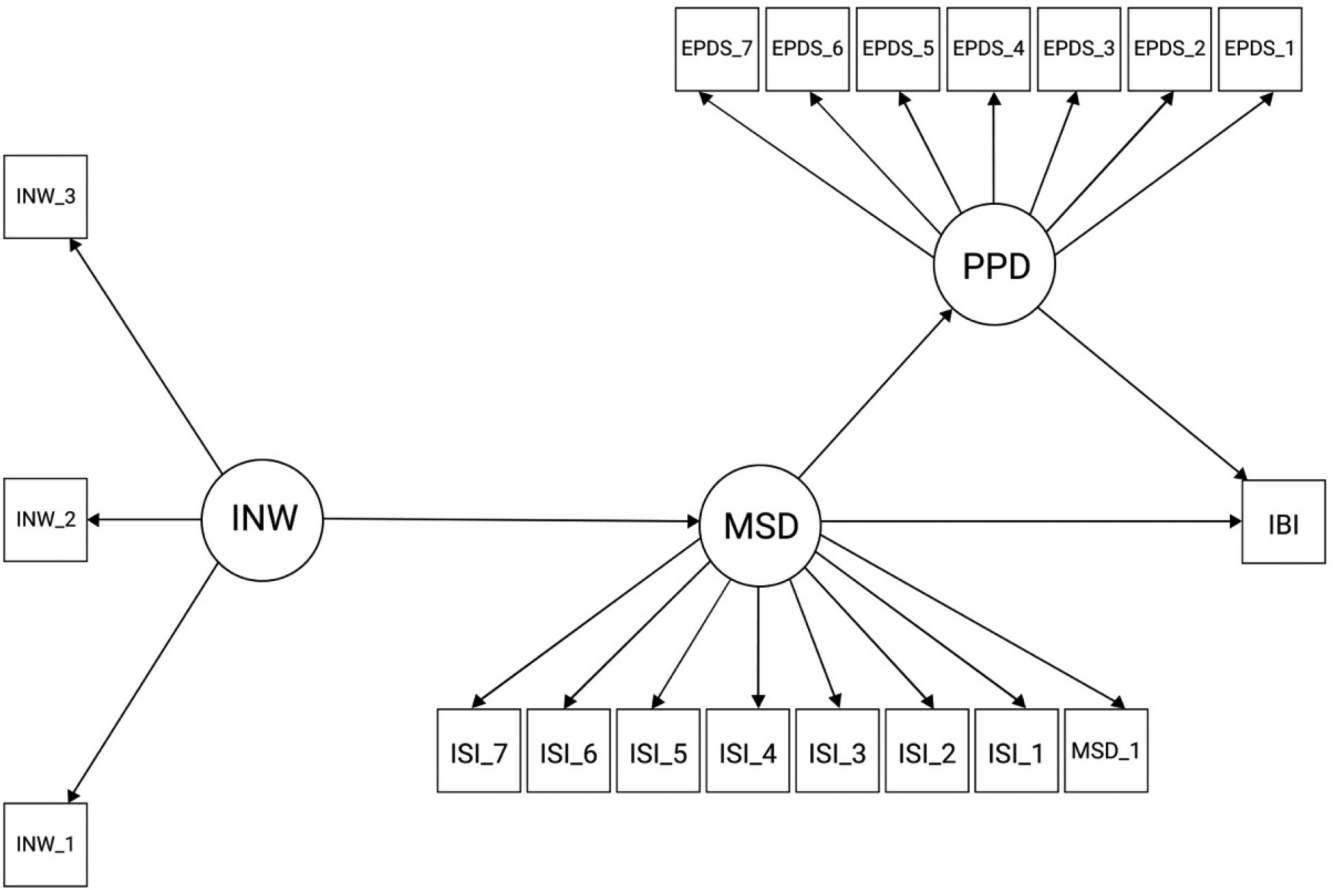
Model visualizing the structural regressions. *Note*. The latent factors are represented by circles, whereas their indicators and the separate item for interbirth interval (IBI) are represented by squares. IBI is regressed on postpartum depression symptoms (PPD) and maternal sleep disturbance (MSD), postpartum depression symptoms are regressed on maternal sleep disturbance, and maternal sleep disturbance is regressed on infant night waking (INW). The residual correlations between variables and the residual variance of the individual variables are not visualized. EPDS  =  seven-item short version of the Edinburgh Postnatal Depression Scale (Cox et al., 1987; Gollan et al., 2017); ISI  =  the Insomnia Severity Index (Morin et al., 2011). The INW items and MSD_1 were self-constructed.

As a preliminary step, we calculated zero-order Spearman correlations between items and between composite variables of the study measures. We visualized the correlations using the *corrplot* package (Wei & Simko, 2017) in *R* (version 3.5.0; R Core Team, 2018). We then conducted structural regressions separately for the two first children at two age intervals (0–1 years; 1–3 years) using the *lavaan* package (Rosseel, 2012). Figure 1 visualizes the structural regression models. According to our hypotheses, we assumed that infant night waking would be positively associated with maternal sleep disturbance, and that maternal sleep disturbance in turn would be associated with postpartum depression symptoms and longer IBIs. We further explored whether the hypothesized association between maternal sleep disturbance and longer IBIs was mediated by postpartum depression symptoms.

For the two structural regressions conducted at the age interval 1–3 years, we removed all participants that had already had an additional child within three years of the focal child. This was done to make sure that infant night waking, maternal sleep disturbance, and postpartum depression symptoms were not due to the additional child. To investigate whether any outliers influenced the results, we additionally analyzed the sample with winsorized IBIs by constraining the length of the 5% longest IBIs to 101 months (i.e., the cutoff of the 95% shortest IBIs).

## Results

### Descriptive Results

Descriptive statistics of the sample are presented in Table 1.

**Table 1.** Sample Descriptives.

Variables	*n*	%
First child’s sex		
Female	358	49.1
Male	368	50.5
Other	3	0.4
Second child’s sex		
Female	347	47.6
Male	379	52
Other	3	0.4
Relationship status		
Married/cohabiting with (one of) the children’s biological father	545	74.8
In a relationship with (one of) the children’s biological father	5	0.7
Married/cohabiting with someone other than the children’s biological father	42	5.8
In a relationship with someone other than the children’s biological father	47	6.4
Single	90	12.3
Same biological father^a^		
All of the children	611	83.9
First and second child, not third child	32	4.4
Second and third child, not first child	32	4.4
First and third child, not second child	1	0.1
None of the children	52	7.1
Breastfeeding, yes^b^		
First child	660	90.5
Second child	286	96.6
Use of contraceptives, yes		
First IBI	416	57.1
Second IBI	163	55.1
Miscarriage, yes^c^		
First IBI	118	16.2
Second IBI	41	13.9
Abortion, yes		
First IBI	22	3
Second IBI	12	4.1
In-vitro fertilization		
For first child	21	2.9
For second child	18	2.5
For third child	8	2.7^d^
For any of the children	31	4.3

*Note. n*  =  729. IBI  =  interbirth interval.

^a^ One participant’s response was excluded due to an illogical answer (*n*  =  728).

^b^ Mean duration for the first child was 11.0 months (*SD*  =  7.7) and for the second child 11.5 months (*SD*  =  7.6).

^c^ Mean number of miscarriages for the first IBI was 1.3 (*SD*  =  0.9) and for the second IBI 1.2 (*SD*  =  0.5).

^d^ Represents the percentage of mothers with three or more children (*n*  =  296).

Of the participating mothers (*n*  =  729), 433 (59.4%) had two children and 296 (40.6%) had three or more. The mean for the IBI between the first and second child was 39.9 months (*SD*  =  29.1) and for the IBI between the second and third child 46.4 months (*SD*  =  33.5).

Means for the different children and age groups for the composite variables indicating infant night waking, maternal sleep disturbance, and postpartum depression symptoms illustrate mean levels for the sum variables and are presented in Table 2. Note that the regressions used latent variables.

**Table 2.** Means for the Different Children and Age Groups for the Composite Variables Infant Night Waking, Maternal Sleep Disturbance, and Postpartum Depression Symptoms.

	Child	Age (years)	*M*	*SD*
Infant night waking	1st	0–1	9.1	3.5
		1–3	6.3	2.8
	2nd	0–1	8.9	3.3
		1–3	6.8	3.2
Maternal sleep disturbance	1st	0–1	19.9	7.8
		1–3	15.5	6.5
	2nd	0–1	22.4	7.9
		1–3	19.8	7.3
Postpartum depression symptoms	1st	0–1	5.2	4.5
		1–3	4.2	4.1
	2nd	0–1	5.1	4.6
		1–3	4.2	4.1

*Note.* Higher scores indicate more infant night waking, maternal sleep disturbance, and postpartum depression symptoms. For assessing infant night waking, we used three self-constructed questions with the range 3–15. For assessing maternal sleep disturbance, we used one self-constructed question, as well as the Insomnia Severity Index (Morin, 1993) with the range 0–28, with higher scores indicating more sleep disturbance. For the self-constructed question, that measured average hours of sleep, we set the outliers to 12 and reverse-coded the item, giving it a range of 0–12 with higher scores indicating more problems. Thus, the final range for maternal sleep disturbance was 0–40. To assess postpartum depression symptoms, we used the seven-item short version of the Edinburgh Postnatal Depression Scale (Cox et al., 1987; Gollan et al., 2017), with the range 0–21, with higher scores indicating more postpartum depression symptoms.

### Correlations

**Figure 2.**
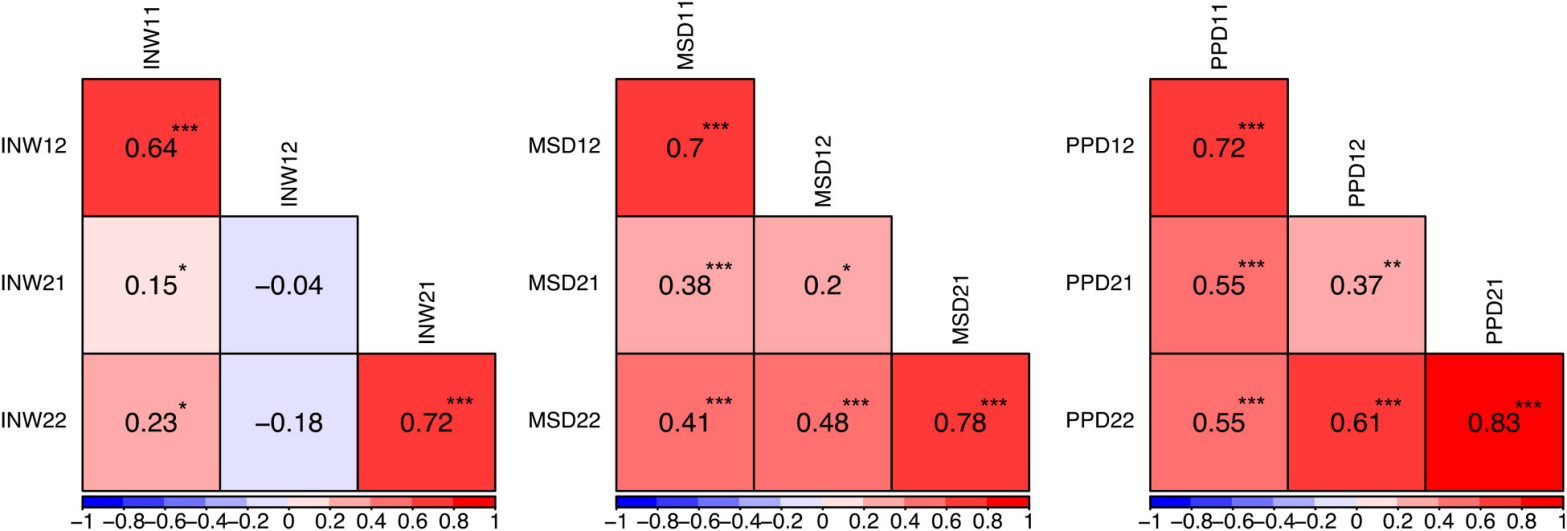
Plots of the zero-order correlations of the composite study variables. *Note*. INW = infant night waking, MSD = maternal sleep disturbance, and PPD = postpartum depression symptoms. Positive correlations are shown in red, negative correlations are shown in blue, and correlations close to zero are shown in white. The first number following the abbreviation stands for either the first child (=1) or the second child (=2), with the second number representing the age interval in question (1  =  0–1 years, 2  =  1–3 years). *** <.001, ** <.01, * <.05.

Correlations of the composite variables indicating infant night waking, maternal sleep disturbance, and postpartum depression symptoms are shown in Figure 2. For all composite variables, the cross-time, within-subject correlations (i.e., for the same child during the two age intervals) were strong and positive. The within-time, between-subject correlations (i.e., for the first and the second child during the same age interval) for maternal sleep disturbance and postpartum depression symptoms were also positive. For infant night waking, however, the correlations between the first child at the second age interval (1–3 years) and the second child at both age intervals were negative.

Correlations for individual items are presented in Supplementary Online Figure 1. For both children at the first age interval (0–1 years), IBI was significantly and positively correlated with all but one of the maternal sleep disturbance items, indicating that mothers with more sleeping problems had longer IBIs. The effects, however, were small (*r*  =  .01–.05). For the first child at the second age interval (1–3 years), the associations were in the opposite direction: IBI was significantly and negatively associated with all maternal sleep disturbance items (*r*  =  -.03– -.16), indicating that mothers with more sleeping problems had shorter IBIs. For the second child at the second age interval, some associations between IBI and maternal sleep disturbance were positive, and some were negative.

### Results From the Structural Regression Model

**Measurement model.** We specified a model with three latent variables, *Infant Night Waking* (three indicators), *Maternal Sleep Disturbance* (eight indicators), and *Postpartum Depression Symptoms* (seven indicators). For the measurement model, responses for both children at both age intervals were included. Our initial model showed suboptimal fit (χ^2^ [132]  =  2171.165, *p* < .001, CFI  =  .975, TLI  =  .971, RMSEA  =  .104 [.100, .108], SRMS  =  .052), so we adjusted the model by adding five residual correlations, all within scales. The residual correlations increased the χ^2^ fit with at least 50 points. Two of the residual correlations were between EPDS items (1 and 2, measuring the mother’s ability to see the funny side of things and ability to enjoy future events; 5 and 6, measuring the mother’s level of misery and unhappiness). Three of the residual correlations were between ISI items (1 and 2, measuring problems falling and staying asleep; 2 and 3, measuring problems staying asleep and waking up too early; and 1 and 3, measuring problems falling asleep and waking up too early). After the above-mentioned adjustments, our final model showed adequate fit (χ^2^ [127]  =  1254.443, *p* < .001, CFI  =  .986, TLI  =  .983, RMSEA  =  .079 [.075, .083], SRMS  =  .042).

**Structural regressions.** The results of the four structural regressions are summarized in Table 3. The winsorized analyses showed that the results were not influenced by any outliers (see Supplementary Online Table 1).

**Table 3.** Results From the Structural Regression Models.

Child	Age	*n*		*b*	β	95% CI_β_	*SE*_β_	*z*_β_	*p*
1st	0–1	729	INW → MSD	0.69	.84	[.82; .87]	0.02	33.69	<.001
			MSD → PPD	0.90	.74	[.70; .78]	0.04	24.67	<.001
			PPD → IBI	7.67	.23	[.08; .38]	2.54	3.02	.003
			MSD → IBI	−8.68	−.22	[−.37; −.06]	3.15	−2.75	.006
			Total effect	−1.80	−.05	[−.12; .04]	1.63	−1.11	.269
			Indirect effect	6.88	.17	[.06; .28]	2.30	2.99	.003
1st	1–3	267	INW → MSD	0.62	.78	[.74; .83]	0.04	17.51	<.001
			MSD → PPD	0.83	.69	[.62; .76]	0.06	12.97	<.001
			PPD → IBI	11.15	.28	[.05; .51]	4.69	2.38	.017
			MSD → IBI	−14.45	−.30	[−.53; −.07]	5.71	−2.53	.011
			Total effect	−5.15	−.11	[−.23; .02]	3.11	−1.65	.098
			Indirect effect	9.30	.19	[.04; .35]	3.92	2.37	.018
2nd	0–1	296	INW → MSD	0.68	.82	[.79; .86]	0.03	23.41	<.001
			MSD → PPD	1.01	.78	[.73; .84]	0.05	20.02	<.001
			PPD → IBI	−1.68	−.05	[−.29; .20]	4.38	−0.38	.701
			MSD → IBI	3.02	.07	[−.17; .30]	5.46	0.55	.581
			Total effect	1.32	.03	[−.09; .15]	2.67	0.49	.621
			Indirect effect	−1.70	−.04	[−.23; .15]	4.41	−0.38	.701
2nd	1–3	137	INW → MSD	0.56	.81	[.74; .87]	0.05	12.32	<.001
			MSD → PPD	1.15	.81	[.76; .87]	0.09	12.78	<.001
			PPD → IBI	9.89	.27	[−.09; .64]	6.70	1.48	.140
			MSD → IBI	−13.09	−.25	[−.63; .12]	9.95	−1.32	.188
			Total effect	−1.70	−.03	[−.21; .15]	4.74	−0.36	.720
			Indirect effect	11.40	.22	[−.08; .52]	8.02	1.42	.156

*Note.* INW  =  infant night waking; MSD  =  maternal sleep disturbance; PPD  =  postpartum depression symptoms; IBI  =  interbirth interval. Total effect  =  Effect of MSD on IBI (either mediated through PPD or alone). Indirect effect  =  Proportion of association between MSD and IBI mediated by PPD.

The results for the structural regressions showed that infant night waking was associated with maternal sleep disturbance for both children at both age intervals. Maternal sleep disturbance was also associated with postpartum depression symptoms for both children at both age intervals. Interestingly, postpartum depression symptoms were associated with longer IBIs for the first child during both age intervals but were not significantly associated with longer IBIs for the second child at either age interval. Finally, maternal sleep disturbance was associated with shorter IBIs for the first child at both age intervals, but not significantly associated with IBIs for the second child at either age interval. The total effect of maternal sleep disturbance (i.e., either mediated through postpartum depression symptoms or alone) was not statistically significant in any of the regressions. The indirect effect (i.e., the proportion of the association between maternal sleep disturbance and IBIs mediated by postpartum depression symptoms) was statistically significant and positive for the first child during the first year, but not for the first child at the second age interval or the second child at either age interval.

## Discussion

Based on the premise that infant night waking is an adaptation to increase IBIs by exhausting the mother (Gunst et al., 2021), the present study served as an initial attempt at examining whether maternal sleep disturbance was associated with longer IBIs. We also explored whether the association between maternal sleep disturbance and IBI length would be mediated by postpartum depression symptoms.

Completely in line with our expectations based on previous research, we found that infant night waking was associated with more maternal sleep disturbance and that maternal sleep disturbance was associated with more postpartum depression symptoms.

In the zero-order correlation analysis (that is, before including postpartum depression symptoms in the latent regression model) there were weak positive correlations between IBI length and the maternal sleep disturbance items during the first year postpartum, indicating that mothers with more sleeping problems had slightly longer IBIs. However, in the structural regression analysis, we found that maternal sleep disturbance—when controlled for postpartum depression symptoms—was associated with *shorter* IBIs for the first child at both age intervals. This indicates that first-time mothers who suffer from more severe sleep problems, but not from symptoms of postpartum depression, have a shorter IBI between their first and second child. These results contradict our prediction that postpartum depression symptoms could be an unintentional byproduct of the infant’s behavior to exhaust their mother. A potential reason for this association could be received social support. Previous research has theorized that postpartum depression signals the need for additional social support (Hagen, 1999). It is possible that mothers who are sleep deprived but receive social support do not develop postpartum depression symptoms, and this protects against long IBIs. It is also more likely that these mothers have a supportive partner, facilitating the conception of an additional child. Another explanation for this result could be that mothers who had children in quick succession remembered the first years postpartum as a time when they were constantly tired. The memory of being constantly tired could make it more likely that they report more severe maternal sleep disturbance.

Postpartum depression symptoms predicted longer IBIs for the first child at both age intervals. These results support the notion that postpartum depression in and of itself could be viewed as adaptive from the offspring’s fitness’ point of view, since more severe postpartum depression symptoms are associated with longer IBIs. Furthermore, the indirect effect of maternal sleep disturbance on IBIs via postpartum depression symptoms indicates that postpartum depression symptoms indeed mediate part of the association between maternal sleep disturbance and IBIs for the first child during the first age interval. However, the total effect of maternal sleep disturbance (either mediated through postpartum depression symptoms or alone) on IBIs was not significant for any of the regressions. That is, accounting for the association between postpartum depression symptoms and longer IBIs, maternal sleep disturbance did not predict shorter IBIs any longer (i.e., inconsistent/competitive mediation).

Finally, the results imply that there is a difference between mothers of two and mothers of three or more children. For both the associations between maternal sleep disturbance and IBIs as well as for postpartum depression symptoms and IBIs, there were no significant associations for the second child at either age interval. The average age to give birth for first-time mothers in Finland was 29.3 years in 2018 (Terveyden ja hyvinvoinnin laitos, 2020). This is relatively late, considering the human female’s fertility markedly decreases in the mid-30s (Liu et al., 2011). Considering the high age for first-time mothers in Finland, women who want three or more children are forced to have them in relatively rapid succession due to the decreased fertility. This could mean that cultural constrains can modify the association between maternal sleep problems and IBI length, providing a potential explanation for the nonsignificant results for the second IBI.

### Limitations

The present study is one of the first to study the relationship between maternal sleep and IBI length. However, there are several aspects that need to be considered before drawing strong conclusions from the associations found. One limitation of the current study is that data were gathered retrospectively, as it increases both under- and overreporting symptoms and behaviors. The high, positive within-subject cross-time correlations could be a sign that it was hard for the mothers to differentiate between their experiences during the two age intervals. As being able to measure an IBI per definition requires that some time has passed since the maternal sleep disturbance and postpartum depression of interest, a longitudinal design could minimize retrospective bias. Moreover, one study inclusion criterion was having at least two children, excluding any mothers that decided not to have additional children after the first child due to severe postpartum depression or sleeping difficulties. All mothers who had an IBI shorter than three years were also excluded from the analyses for the second age interval (1–3 years), skewing the results in favor of longer IBIs. The sample size for these analyses was also smaller, resulting in issues with statistical power.

Finally, the participants in our study were in large part recruited via a social media ad targeting women living in Finland. The Nordic countries stand out with generous social support and parental leave policies (Pronzato, 2009)—possibly reducing the generalizability of the results.

## Conclusions

Contrary to our initial hypothesis, we did not find support for the notion that infant night waking is an adaptive strategy in order to tire the mother, making her less likely of conceiving and gestating an additional child, thus lengthening the IBI. Interestingly, the results indicate that postpartum depression symptoms could themselves be adaptive from the offspring’s fitness’ point of view, by being associated with longer IBIs. In other words, if infant night waking was indeed an adaptive strategy to extend the IBI by exhausting the mother, it seems like maternal sleeping difficulties alone are not sufficient to result in longer IBIs. Future research should investigate the role of maternal social support to shed light on potential explanations as to why mothers who suffer from sleep disturbance but not from postpartum depression have shorter IBIs. Furthermore, investigation into the difference between mothers of two and mothers of three or more children is needed to understand why some of our results were significant only for the first IBI.

## Supplemental Material

sj-docx-1-evp-10.1177_14747049211046162 - Supplemental material for The Mother–Offspring Conflict: The Association Between Maternal Sleep, Postpartum Depression, and Interbirth Interval Length Click here for additional data file. Supplemental material, sj-docx-1-evp-10.1177_14747049211046162 for The Mother–Offspring Conflict: The Association Between Maternal Sleep, Postpartum Depression, and Interbirth Interval Length by Annika Gunst, Elin Sjöström, My Sundén and Jan Antfolk in Evolutionary Psychology

sj-docx-2-evp-10.1177_14747049211046162 - Supplemental material for The Mother–Offspring Conflict: The Association Between Maternal Sleep, Postpartum Depression, and Interbirth Interval Length Click here for additional data file. Supplemental material, sj-docx-2-evp-10.1177_14747049211046162 for The Mother–Offspring Conflict: The Association Between Maternal Sleep, Postpartum Depression, and Interbirth Interval Length by Annika Gunst, Elin Sjöström, My Sundén and Jan Antfolk in Evolutionary Psychology
