# Supplementary material for: The Mother–Offspring Conflict: The Association Between Maternal Sleep, Postpartum Depression, and Interbirth Interval Length
Source: Evol Psychol. 2021 Oct 12;19(4):14747049211046162. doi: 10.1177/14747049211046162 (PMC10358409; doi:10.1177/14747049211046162)
Supplement: sj-docx-1-evp-10.1177_14747049211046162 - Supplemental material for The Mother–Offspring Conflict: The Association Between Maternal Sleep, Postpartum Depression, and Interbirth Interval Length [file sj-docx-1-evp-10.1177_14747049211046162.docx]

| **Supplementary Online Table 1**  *Results from the Winsorized Structural Regression Models* | | | | | | | | | |
| --- | --- | --- | --- | --- | --- | --- | --- | --- | --- |
| Child | Age | *n* |  | *b* | β | 95% CI_β_ | *SE*_β_ | *z*_β_ | *p* |
| 1^st^ | 0–1 | 729 | INW 🡪 MSD | 0.69 | .84 | [.79; .90] | 0.03 | 30.67 | < .001 |
|  |  |  | MSD 🡪 PPD | 0.90 | .74 | [.67; .79] | 0.03 | 28.52 | < .001 |
|  | |  | PPD 🡪 IBI | 5.62 | .23 | [.09; .36] | 0.07 | 3.33 | .001 |
|  | |  | MSD 🡪 IBI | -5.91 | -.20 | [-.33; -.06] | 0.07 | -2.86 | .004 |
| 1^st^ | 1–3 | 267 | INW 🡪 MSD | 0.62 | .78 | [.67; .90] | 0.06 | 13.15 | < .001 |
|  |  |  | MSD 🡪 PPD | 0.83 | .69 | [.57; .80] | 0.06 | 11.95 | < .001 |
|  | |  | PPD 🡪 IBI | 6.58 | .27 | [.07; .47] | 0.10 | 2.67 | .008 |
|  | |  | MSD 🡪 IBI | -9.16 | -.31 | [-.52; -.11] | 0.11 | -2.97 | .003 |
| 2^nd^ | 0–1 | 296 | INW 🡪 MSD | 0.68 | .82 | [.68; .96] | 0.07 | 11.50 | < .001 |
|  |  |  | MSD 🡪 PPD | 1.01 | .78 | [.67; .90] | 0.06 | 13.65 | < .001 |
|  | |  | PPD 🡪 IBI | -1.76 | -.06 | [-.30; .17] | 0.12 | -0.54 | .588 |
|  | |  | MSD 🡪 IBI | 2.88 | .08 | [-.15; .31] | 0.12 | 0.71 | .479 |
| 2^nd^ | 1–3 | 137 | INW 🡪 MSD | 0.56 | .81 | [.59; 1.02] | 0.11 | 7.41 | < .001 |
|  | |  | MSD 🡪 PPD | 1.15 | .81 | [.67; .95] | 0.07 | 11.25 | < .001 |
|  | |  | PPD 🡪 IBI | 3.87 | .17 | [-.20; .54] | 0.19 | 0.91 | .364 |
|  | |  | MSD 🡪 IBI | -4.89 | -.15 | [-.52; .21] | 0.19 | -0.81 | .418 |
| Note. INW = infant night waking; MSD = maternal sleep disturbance; PPD = postpartum depression symptoms; IBI = interbirth interval. | | | | | | | | | |
