# Supplementary material for: The Mother–Offspring Conflict: The Association Between Maternal Sleep, Postpartum Depression, and Interbirth Interval Length
Source: Evol Psychol. 2021 Oct 12;19(4):14747049211046162. doi: 10.1177/14747049211046162 (PMC10358409; doi:10.1177/14747049211046162)
Supplement: sj-docx-2-evp-10.1177_14747049211046162 - Supplemental material for The Mother–Offspring Conflict: The Association Between Maternal Sleep, Postpartum Depression, and Interbirth Interval Length [file sj-docx-2-evp-10.1177_14747049211046162.docx]

**Supplementary Online Figure 1**

Item Correlations Separately for the Two First Children at Two Age Intervals

First Child, 0–1 Years

First Child, 1–3 Years

Second Child, 1–3 Years

Second Child, 0–1 Years

Note. IBI = interbirth interval, INW = infant night waking (self-constructed), MSD = maternal sleep disturbance (self-constructed), ISI = The Insomnia Severity Index (Morin, 1993). EPDS = The Edinburgh Postnatal Depression Scale short form (Gollan et al., 2017). Strong positive correlations are shown in red, strong negative correlations are shown in blue, and correlations close to zero are shown in white.
